# Longitudinal ECG changes in tetralogy of Fallot and association with surgical repair

**DOI:** 10.3389/fcvm.2024.1349166

**Published:** 2024-03-28

**Authors:** Misha Bhat, Torsten Malm, Gunnar Sjöberg, Felicia Nordenstam, Katarina Hanséus, Carl-Johan Rosenkvist, Petru Liuba

**Affiliations:** ^1^ Department of Pediatric Cardiology, Pediatric Heart Center, Skane University Hospital, Lund, Sweden; ^2^ Department of Clinical Sciences, Lund University, Lund, Sweden; ^3^ Department of Pediatric Cardiac Surgery, Pediatric Heart Center, Skane University Hospital, Lund, Sweden; ^4^ Department of Pediatric Cardiology, Department of Women’s and Children’s Health Karolinska Institutet, Karolinska University Hospital, Stockholm, Sweden; ^5^ Department of Pediatrics, Kalmar Länssjukhus, Kalmar, Sweden

**Keywords:** tetralogy of Fallot, trans-annular patch, valve sparing surgery, electrocardiography, QRS duration, QTc, fragmentation, monocusp pulmonary valve

## Abstract

**Background:**

ECG abnormalities have been linked to adverse changes in right ventricular (RV) morphology and poor clinical outcomes in repaired Tetralogy of Fallot (rTOF). Our aim was to describe how ECG changes progress in early and intermediate follow-up and whether types of surgical strategy at the time of primary repair affected these changes.

**Methods:**

We studied patients with rTOF born 2000–2018 operated at our institution. Seven time points in relation to primary repair, follow-up, and pulmonary valve replacement (PVR) were identified. Patients correct with valve sparing repair (VSR), trans-annular patch (TAP) including with a monocusp valve (TAP + M) and with at least 3 ECGs were included. PQ interval, QRS duration, dispersion, and fragmentation, QTc duration and dispersion, JTc as well as presence of a right bundle branch block (RBBB) were analyzed. Medical records were reviewed for demographic and surgical data.

**Results:**

Two hundred nineteen patients with 882 ECGs were analyzed with a median follow-up time of 12.3 years (8.4, 17) with 41 (19%) needing PVR during the study period. QRS duration increased at time of primary repair to discharge from 66 msec (IQR 12) to 129 msec (IQR 27) (*p* < 0.0001) and at 1- and 6- year follow-up but showed only a modest and temporary decrease after PVR. QTc increased at the time of primary repair as well as prior to PVR. PQ interval showed a small increase at the time of primary repair, was at its highest prior to PVR and decreased with PVR. Type of surgical repair affected mainly QTc and JTc and was consistently longer in the TAP + M group until PVR. In VSR, QTc and JTc were prolonged initially compared to TAP but were similar after 1 year. After PVR, there were no differences in adverse ECG changes between surgical groups.

**Conclusions:**

PQ interval and QRS duration best correspond to the assumed volume load whereas the relationship with QTc and JTc is more complex, suggesting that these represent more complex remodeling of the myocardium. Before PVR, QTc and JTc are longer in the TAP + M group which may be due to a longer surgical incision.

## 1 Introduction

Tetralogy of Fallot (TOF) is a common major congenital heart defect (CHD) with an incidence of 0.35/1,000 live births (1). Short- and long-term survival of repaired tetralogy of Fallot (rTOF) is excellent but there is a significant burden of residual lesions. These include, but are not limited to, residual pulmonary stenosis, pulmonary valve insufficiency, and ventricular dyssynchrony (2). This group of patients is at risk over time of needing reintervention, in particular pulmonary valve replacement, as well as of arrhythmias and sudden cardiac death (3–5). Most patients end up with right bundle branch block (RBBB) following repair (6, 7). Electrocardiogram (ECG) based biomarkers, such as QRS duration, QRS fragmentation (fQRS) as well as dispersion have been showed to be associated with an increased risk for adverse outcomes in rTOF including arrhythmias and sudden cardiac death (8–10). QRS duration is also a marker of electromechanical dissociation, which may be central in the pathogenesis of right ventricular dysfunction and dilatation in certain patients (11, 12). Prolongation of the PR interval is also an emerging marker of RV dysfunction and is associated with an increased risk of arrhythmias (13, 14). Data is equivocal whether these ECG markers are associated with volume load, ventricular dilation, dysfunction or dyssynchrony, whether they are reversible with PVR and there is limited data on association between surgical strategy at primary repair and adverse ECG changes. In the primary repair, using a transannular patch (TAP) may involve a longer incision in the right ventricular outflow tract (RVOT) and leaves the patient with pulmonary regurgitation. This can be temporarily alleviated using a monocusp valve. A valve sparing repair (VSR) may be more likely to involve extensive subvalvular resection and have residual outflow tract obstruction. These differences in technique and sequelae may result in different ECG changes with potentially increased long-term risk.

We sought to describe ECG changes in rTOF over time in a cohort of infant repair, how they change in relation to primary repair, in follow-up and follow-up after PVR and whether type of primary repair is associated with differences in adverse ECG changes.

## 2 Methods

### 2.1 Study design and patient selection

This was a retrospective review of all patients who underwent primary repair of TOF at our institution between 2000 and 2018. Patients with the TOF variants of pulmonary atresia or absent pulmonary valve were excluded as well as other highly complex variants that resulted in single ventricle physiology and TOF combined with a common atrioventricular canal. Patients with minor-moderate associated anomalies such as partial anomalous pulmonary venous return, atrial septal defects and vascular rings were included. Subjects were also excluded if they underwent primary repair with a conduit, if medical records were unavailable, or fewer than 3 ECGs were available for review. Patients were identified through the national Swedish database for CHD (SWEDCON) as well as local surgical databases. Data was entered in an anonymized manner into a REDCAP database hosted at Lund University (15).

### 2.2 Clinical data

Medical records and the national CHD database were reviewed for clinical data including age at diagnosis, gestational age, gender, previous surgery with an eventual aorto-pulmonary shunt, genetic abnormalities, details of the surgical strategy at the time of primary repair as well as any cardiac reoperations. Reoperations were defined as any repeat surgical intervention of the RVOT including PVR. Prematurity was defined as delivery prior to 37 + 0 gestational weeks. Pulmonary valve *Z*-score was calculated using published Pediatric Heart Network data using preoperative echo-measurements (16).

### 2.3 Electrocardiographic analysis

Only standard 12 lead ECGs with a speed of 50 mm/s and of sufficient quality for analysis were included. All measurements were performed manually by a single observer blinded to outcomes and surgical strategy at the time of analysis. Electrocardiograms were analyzed at 7 different timepoints: (1) Prior to primary repair, (2) at discharge from primary repair, (3) at 1 year ± 6 months after primary repair, (4) 6 years ± 1 year from repairs if no previous PVR had been performed, (5) prior to PVR, (6) at discharge from PVR and (7) 1 year ± 6 months after PVR.

The electrocardiographic variables registered were heart rate, QRS angle, PQ-interval, QRS duration, QT interval and QTc, QRS dispersion, QTc and QT dispersion, QRS fragmentation and presence of a bundle branch block. The end of the T-wave was defined according to the tangent method and Bazett's formula was used to calculate QTc (17, 18). JT was calculated by subtracting the QRS duration from the QT and JTc was calculated by subtracting the QRS duration from the QTc. Dispersion was defined as the difference between the maximum and minimum values of QRS and QT respectively in between any of the 12 leads (19). Fragmentation was defined as additional notches in the QRS and severity was classified depending on the number of consecutive leads affected and registered as none (0–1 leads), mild (2–3) moderate (4) vs. severe (≥5) (20).

### 2.4 Statistical analysis

Descriptive statistics were used for demographic and surgical information using *n* (%) for frequencies, mean (standard deviation) for parametric variables and median (interquartile range) for non-parametric variables. For statistical analysis, fragmentation was divided into ≤mild (none or mild) vs. > mild (moderate or severe). Demographic data was compared between the different surgical groups using Kruskal-Wallis for continuous data and chi-square for continuous and binary outcomes respectively. For comparing longitudinal changes in continuous data, Wilcoxon's repeated measures was used between each consecutive timepoint for nonparametric variables and ANOVA with Bonferroni posthoc test for parametric variables whereas logistic regression was used for nominal variables. Fragmentation was divided into non-mild vs. moderate-severe for analysis. The cohort was also divided into those who underwent PVR vs. those without PVR. Comparison of ECG variables at timepoint 3 and 4 between the group needing vs. not needing PVR were performed using Wilcoxon rank sum test and chi-square as well as Kaplan Meier curves using Cox regression. In addition, the change in PQ interval, QRS duration and QTc between timepoint 3 and 4 were also compared between the group needing PVR and not needing PVR using Wilcoxon rank sum test. For QRS duration, the group was divided into the top quartile and compared to rate of PVR using chi square and Kaplan Meier curves. Finally, ECG variables were compared between type of surgical strategy at each timepoints using Kruskal-Wallis and chi-square. For comparing rates of surgical PVR and reoperation between the surgical groups at 10 years after surgery, Kaplan Meier curves with log-rank test was used to adjust for temporal changes in surgical strategy.

Statistical significance was set to a *p*-value of 0.05 or less. Statistical analysis was performed using STATA statistical software version 17.0 (College Station, TX). Graphs were plotted using STATA as well as Microsoft Excel for Mac version 16.66.1.

## 3 Results

### 3.1 Patient characteristics and surgical strategy

**Figure 1:**
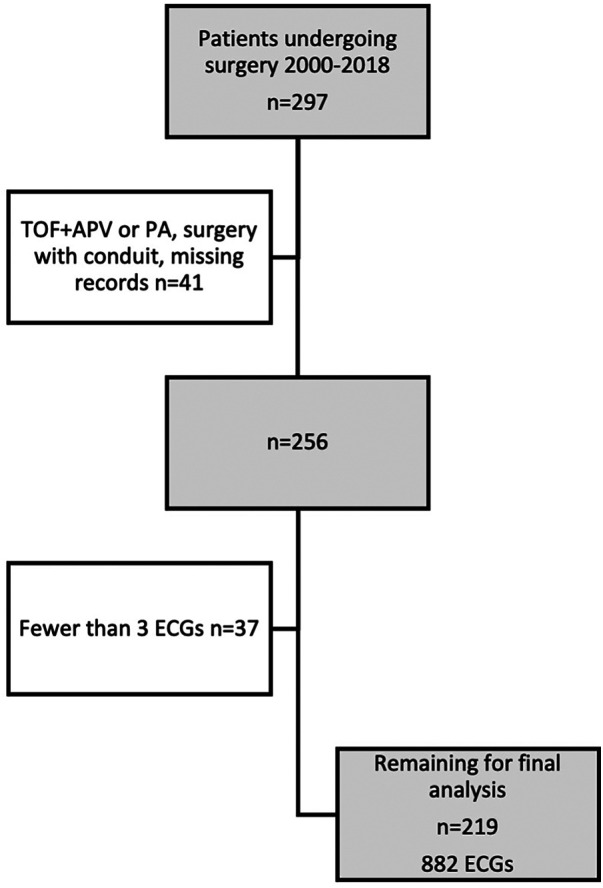
Flowchart showing inclusion and exclusion of subjects. APV, absent pulmonary valve; PA, pulmonary atresia; ECG, electrocardiogram.

A total of 297 patients with TOF were identified in the database. Of these, 41 were excluded based on anatomical variants, surgery with conduit or missing medical records. An additional 37 patients were excluded based upon having less than 3 ECGs of adequate quality available for review, yielding a total of 219 remaining subjects with a total of 882 ECGs for analysis (Figure 1, Table 3).

Median follow up time was 12.3 years (IQR 8.4, 17) with a median age at primary repair at 4.9 months (IQR 3.4, 6.9). All patients underwent trans-atrial repair. Ninety patients (41%) underwent valve sparing repair (VSR) with 48 (22%) and 81 (37%) undergoing repair with trans-annular patch (TAP) and TAP with a monocusp reconstruction (TAP + M) of the pulmonary valve respectively. The median age for PVR was 7.9 years (IQR 4.8, 10.5) and occurred 6.9 (IQR 4.4, 10.2) years after primary repair. The median age for reoperation was 6.0 years (IQR 3.0, 9.9), occurring 5.5 years (IQR 2.7, 9.6) after primary repair.

Demographic and basic surgical data are summarized in Table 1. During the study period, 9 patients had arrythmias. Five patients had SVT (including AVRT), nodal tachycardia and/or JET around the time of primary repair. 2 patients had ventricular tachycardias requiring ablation at 12- and 15-years age, of which one patient required an ICD. Two patients had frequent premature ventricular complexes with onset at 6- and 14-years age respectively.

**Table 1:** Demographic and basic surgical data for entire cohort.

Male gender		134 (61)
Age of follow-up (yrs)		12.3 (8.4, 17)
Prior shunt		45 (21)
Premature		41 (20)
Fetal diagnosis		53 (25)
Median age primary repair (months)		4.9 (3.4, 6.9)
Type of primary repair		
	VSR	90 (41)
	TAP	48 (22)
	TAP + M	81 (37)
Median Z-score of PV		−2.4 (−3.5, −1.1)
Reoperation		57 (26)
PVR		41 (19)

Continuous data are presented as mean ± SD or median (IQR). Categorical data are presented as *n* (%).

VSR, valve sparing repair; TAP, trans-annular patch; TAP + M, trans-annular patch with monocusp reconstruction; PV, pulmonary valve; PVR, pulmonary valve replacement.

In comparing the characteristics of the three surgical strategies there were significant differences in gender (*p* = 0.016) with the highest percentage of males in the TAP group, age at time of study with the longest median follow up in the TAP group (16.4 years; IQR 12.9, 20), and shortest in the TAP + M group (10.5; IQR 7.8, 13.2; *p* = 0.0001) (Table 2). Furthermore there were significant differences in need for prior shunt with the highest rate in the TAP + M group (32% vs. 10% in the VSR group and 21% in the TAP group; *p* = 0.002) and pulmonary valve *Z*-score being the smallest in the TAP + M group (−3.5; IQR −4.3, −2.9) vs. the TAP group (−2.7; IQR −3.4, −2.1) and the VSR group (−1.0; IQR −1.6, 0.6; *p* < 0.001). Rate of reoperation at 10 years was greatest in the TAP group (38% vs. 28% in the TAP + M group and 16% in the VSR groups respectively; *p* = 0.05) and VSR had the lowest rate of PVR (5% vs. 21% in the TAP group and 24% in the TAP + M group; *p* = 0.0006). All PVR were surgical in this cohort. There were no differences in rate of fetal diagnosis, age at primary repair or prematurity between the three groups (Table 2).

**Table 2:** Surgical and demographic data by surgical subtype.

	VSR *N* = 90	TAP *N* = 22	TAP + Monocusp *N* = 81	*p*
Male gender	59 (66)	35 (73)	40 (49)	0.016
Age of follow-up (years)	12.1 (7.2, 17.5)	16.4 (12.9, 20.0)	10.5 (7.8, 13.2)	0.0001
Prior shunt	9 (10)	10 (21)	26 (32)	0.002
Premature	13 (16)	13 (30)	15 (19)	0.183
Fetal diagnosis	21 (23)	7 (16)	25 (31)	0.151
Age at primary repair (months)	4.8 (3.1, 6.8)	5.2 (4.1, 8.4)	4.9 (3.2, 6)	0.0854
*Z*-score of PV	−1.0 (−1.6, −0.6)	−2.7 (−3.4, −2.1)	−3.5 (−4.3, −2.9)	<0.001
Reoperation rate at 10 years	16% (10–26%)	38% (17–43%)	28% 18–41%)	0.05
PVR at 10 years	5% (2–14%)	21% (12–36%)	24% (15–38%)	0.0006

Continuous data are presented as mean ± SD or median (IQR). Categorical data are presented as *n* (%). Reoperation and PVR rates are presented as % (95% CI).

VSR, valve sparing repair; TAP, trans-annular patch; TAP + M, trans-annular patch with monocusp reconstruction; PV, pulmonary valve; PVR, pulmonary valve replacement.

### 3.2 Longitudinal ECG changes

ECG characteristics at each time point are summarized in Table 3.

**Table 3:** Summary of ECG characteristics at each timepoint.

Variable/timepoint	1 (*n* = 209)	2 (*n* = 210)	3 (*n* = 185)	4 (*n* = 168)	5 (*n* = 40)	6 (*n* = 38)	7 (*n* = 32)
Patient age	4.7 (3.2, 7) mo	5.1 (3.8, 7.5) mo	16.6 (13.8, 20.7) mo	6.4 (6.0, 6.9) yrs	8.0 (5.4, 11.3) yrs	8.0 (5.4, 11.5) yrs	8.8 (6.7, 13.7) yrs
Heart rate (bpm)	133 (123, 143)	146 (134, 156)	115 (105, 127)	84 (73, 94)	83 (76, 95)	96 (77, 107)	76 (66, 93)
PQ interval (msec)	112 (102, 124)	120 (103, 134)	116 (106, 128)	133 (120, 147)	141 (123, 156)	129 (121, 141)	130 (120, 154)
QRS axis (degrees)	103 (85, 117)	85 (0, 113)	66 (39, 83)	81 (61, 96)	84 (69, 111)	85 (66, 111)	73 (63, 95)
QRS (msec)	66 (61, 73)	96 (82, 105)	99 (80, 112)	111 (89, 127)	129 (118, 145)	120 (109, 132)	118 (97, 131)
QRS disp (msec)	10 (7, 16)	13 (9, 17)	14 (10, 18)	17 (13, 23)	20 (15, 35)	21 (14, 27)	22 (10, 28)
QTc (msec)	405 (391, 421)	444 (419, 450)	429 (408, 450)	400 (380, 423)	460 (439, 484)	384 (342, 434)	384 (347, 404)
QTc disp (msec)	32 (22, 47)	47 (32, 64)	49 (37, 68)	49 (36, 69)	69 (46, 113)	163 (122, 209)	116 (84, 129)
JTc (msec)	338 ± 25	348 ± 30	334 ± 26	292 ± 32	328 ± 34	262 ± 42	320 ± 35
RBBB	8 (3, 8)	82 (61)	60 (67)	43 (74)	33 (83)	34 (89)	25 (81)
>mild QRS fragmentation	26 (12)	137 (66)	142 (78)	134 (80)	32 (80)	24 (65)	28 (90)

Continuous data are presented as mean ± SD or median (IQR). Categorical data are presented as *n* (%). RBBB, right bundle branch block; disp, dispersion; mo, months; yrs, years.

1. Prior to primary repair, 2. At discharge from primary repair, 3. 12 ± 6 months after primary repair, 4. 6 years ± 1year after primary repair, 5. Prior to pulmonary valve replacement, 6. At discharge from pulmonary valve replacement and 7. At 12 months +/ 6 months after pulmonary valve replacement.

#### 3.2.1 PQ interval

**Figure 2:**
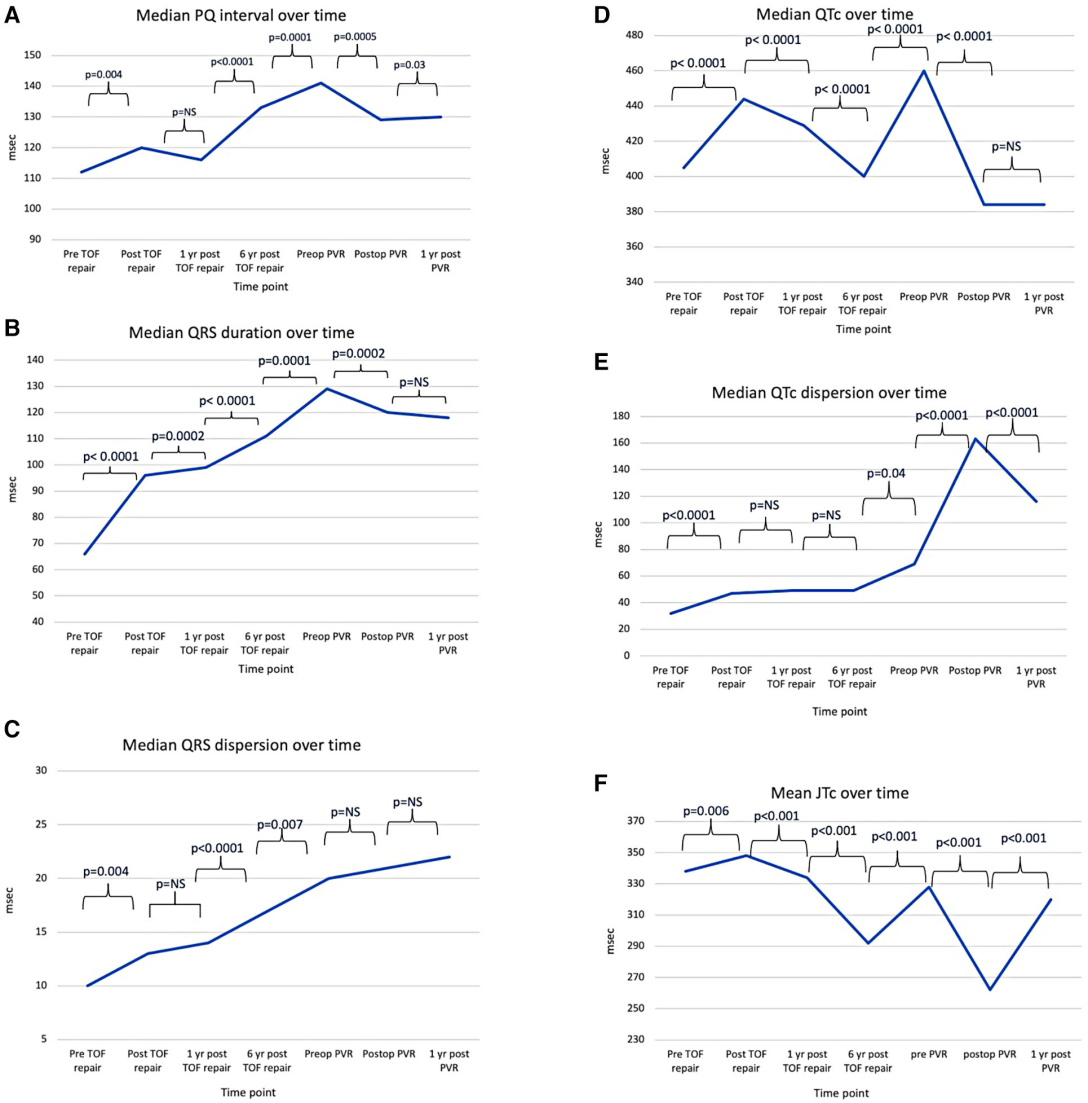
Line charts showing conduction times in ECG at the specified time points. (**A**) Median PQ interval, (**B**) Median QRS, (**C**) Median QRS dispersion, (**D**) Median QTc, (**E**) Median QTc dispersion, (**F**) Mean JTc. TOF, tetralogy of Fallot; PVR, pulmonary valve replacement.

We found an increase in PQ interval at the time of primary repair from 112 (IQR 102, 124) to 120 msec (IQR 103, 134; *p* = 0.004) but not further increase at 1 year follow up (Table 3 and Figure 2A). There was a significant increase in PQ interval from 1- to 6 year follow-up and was highest prior to PVR (141 msec; IQR 123, 156), whereas there was a decrease to discharge from PVR to 129 msec (IQR 121, 141; *p* = 0.0005) with a small increase to the 1 year post PVR time point to 130 msec (IQR 120, 154; *p* = 0.03).

#### 3.2.2 QRS duration and dispersion

There was a significant increase in median QRS duration between each subsequent time point leading up to PVR from 66 msec (IQR 61, 73) prior to primary repair and 129 msec (IQR 118, 145) prior to PVR (Table 3 and Figure 2B). There was a small but significant decrease at the time of discharge from PVR to 120 msec (IQR 109, 132; *p* = 0.002) but no further decrease one year after PVR.

For QRS dispersion there was a small increase at the time of primary repair (*p* = 0.04) with no further increase after one year (Table 3 and Figure 2C). There was, however, further increase at the 6 year time point and prior to PVR (*p* < 0.0001 and 0.007 respectively). Following PVR, there was no significant change in QRS dispersion but a trend of increase.

#### 3.2.3 QTc and QTc dispersion

There was an increase in QTc from prior to primary repair to discharge from primary repair (*p* < 0.0001), whereas there was a continued decrease at 1 year and 6 years post primary repair (*p* < 0.0001 and *p* < 0.0001). (Table 3 and Figure 2D). QTc increased again at prior to PVR (*p* < 0.0001) and decreased at discharge from PVR (*p* < 0.0001) but no further change was seen at 1 year following PVR.

There was a small but significant increase in QTc dispersion from admission to discharge from primary repair (*p* < 0.0001), but no further increase at 1 year or 6 years after primary repair (Table 3 and Figure 2E). QTc dispersion increased further at the PVR (*p* = 0.04) with the largest increase following PVR from 69 (IQR 46, 113) to 163 msec (IQR 122, 209; *p* < 0.0001), but decreased to 116 msec (IQR 84, 129) by 1 year following PVR (*p* < 0.0001).

#### 3.2.4 JTc interval

There were significant differences between each subsequent time point (Table 3, Figure 2F). There was a small increase at the time of primary repair, from 338 ± 25 msec to 348 ± 30 msec (*p* = 0.006) and a decrease to 334 ± 26 msec at one year after primary repair (*p* < 0.001) and further decrease 6 years after primary repair to 292 ± 32 msec (*p* < 0.001). It increased again prior to PVR to 328 ± 24 msec but decreased significantly to 262 ± 42 msec (*p* < 0.001) following PVR but returned to 320 ± 35 msec one year after PVR (*p* < 0.001).

#### 3.2.5 Right bundle branch block

**Figure 3:**
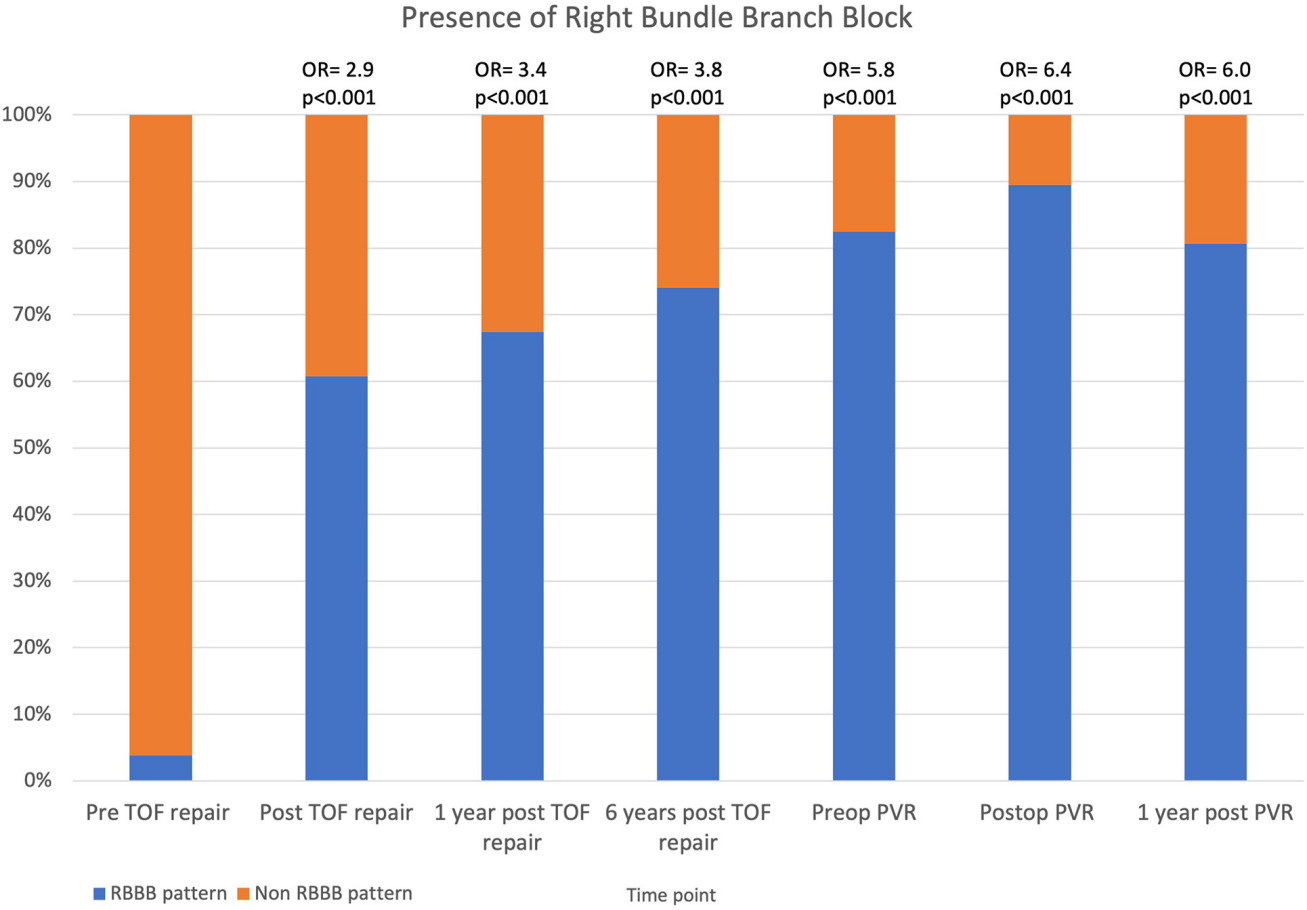
Stacked column chart showing the prevalence of RBBB pattern on ECGs in TOF. Odds ratio at each time point are compared to pre TOF repair. RBBB, right bundle branch block; TOF, tetralogy of Fallot; PVR, pulmonary valve replacement.

Only 3.8% of the patients had a RBBB pattern prior to their primary repair with a large increase after surgery (61%), with a further increase at each time point, peaking before (83%) and at discharge from PVR (89%) (Table 3 and Figure 3). By logistic regression, the presence of RBBB was significantly higher at each time point compared to before primary repair, with the highest OR being at discharge from PVR (89%; OR 6.4; *p* < 0.001).

#### 3.2.6 QRS fragmentation

**Figure 4:**
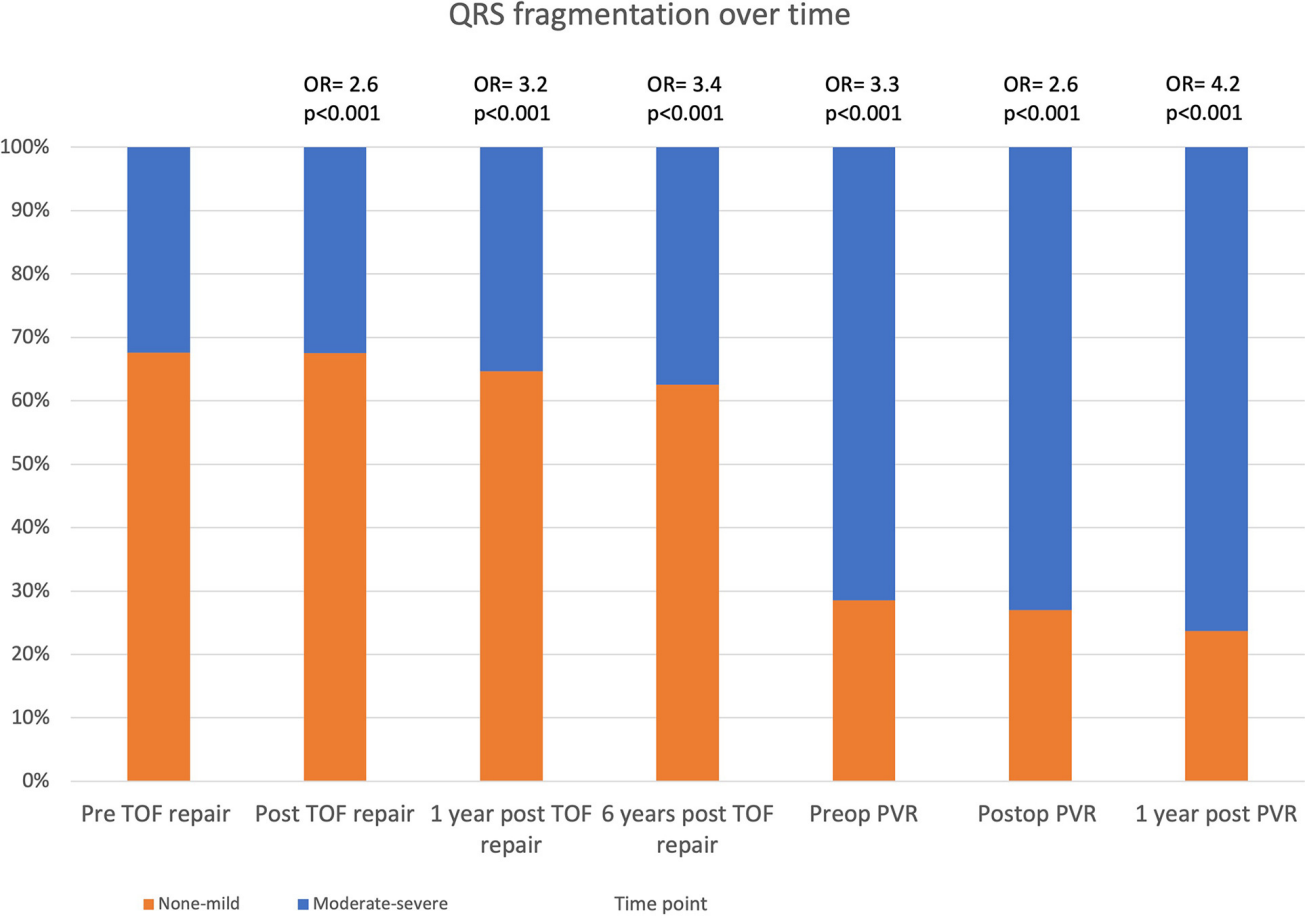
Stacked column chart showing prevalence of >mildly fragmented QRS over time in TOF. Odds ratio at each time point are compared to pre TOF repair. TOF, tetralogy of Fallot; PVR, pulmonary valve replacement.

Only 12% of patients had > mild fQRS prior to primary repair, with a significant increase following surgery to 66% (Table 3 and Figure 4). The odds of having >mild fQRS was greater than prior to surgery at each time point but was greatest 1 year after PVR (90%; OR 4.2; *p* < 0.001).

### 3.3 Variables at 1 year postop and 6 years postop associated with PVR

In comparing ECG variables between the group that underwent PVR and the one that didn't, there were no significant difference in QRS duration at 1 year after primary repair but QRS duration at 6 years was significantly higher in the group that underwent PVR (120 vs. 110 msec; *p* = 0.007) for the whole cohort, independently of type of surgical repair (Table 4).

Having a more than mild QRS fragmentation was more common at 1 year after primary repair in the group undergoing PVR vs. no PVR (93 vs. 75%; *p* = 0.02), but not at 6 years (Table 4).

Those with RBBB at 6 years also have a trend towards higher rates of PVR (86% vs. 72%, HR = 1.3; *p* = 0.06) but not at 1 years follow up.

There were no significant differences between the two groups in terms of PQ interval, QRS dispersion, QTc, QTc dispersion or JTc. There was no significant difference in rate of PVR in the group with the top quartile of QRS duration, as compared to those with shorter QRS.

There were no significant differences in the increase in PQ interval, QRS duration and QTc between timepoint 3 and 4 between the group needing PVR vs. the group that didn't need PVR.

### 3.4 ECG changes by surgical strategy

**Figure 5:**
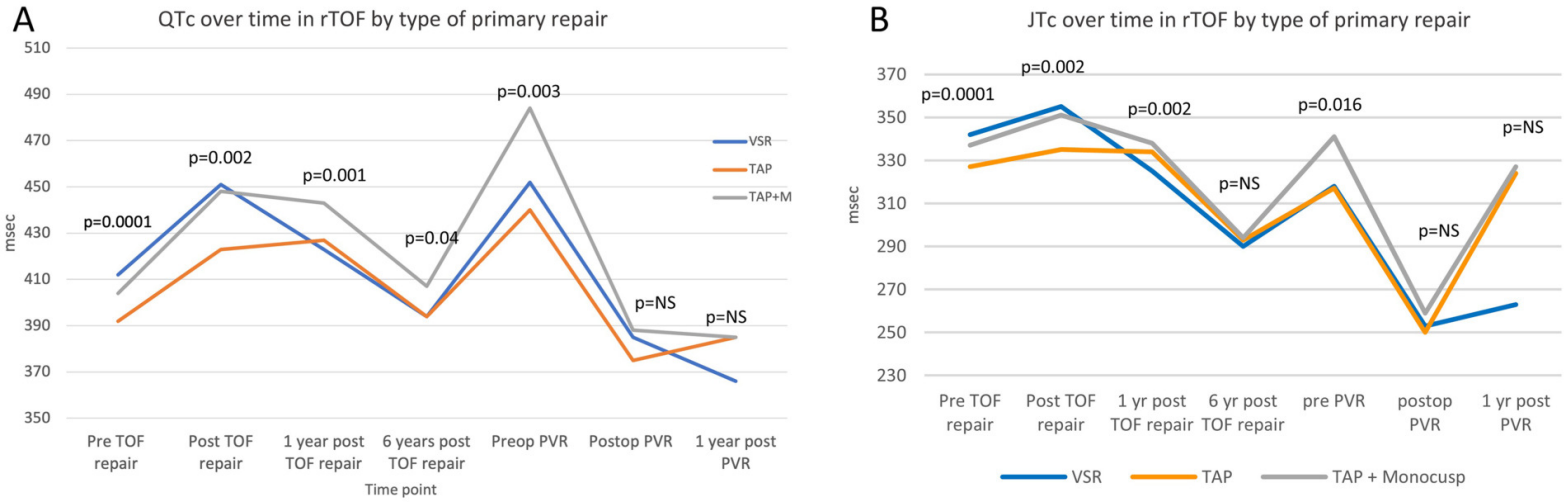
Line diagrams showing comparing QTC (**A**) and JTc (**B**) by type of primary repair. PVR, pulmonary valve replacement; VSR, valve sparing repair; TAP, trans-annular patch; TAP + M, transannular patch with a monocusp reconstruction.

There were no differences in PQ interval, QRS duration, QRS dispersion, presence of RBBB pattern or >mild fragmentation between the three surgical groups (VSR, TAP, TAP + M) at any of the set time points. There was only a significant difference in QTc dispersion at one time-point, namely preoperative (*p* = 0.0018) but no changes at any of the other timepoints. There were, however, significant differences between the surgical groups at multiple time points in terms of QTc and JTc (Table 5, Figures 5A,B).

**Table 4:** Variables associated with pulmonary valve replacement.

Variable	Time point	PVR group	Non PVR group	*P*
QRS duration (msec)	6 years after primary repair	120 (109, 138)	110 (88, 126)	0.007
>mild QRS fragmentation	1 year after primary repair	25 (93%)	117 (75%)	0.04
RBBB	6 years after primary repair	25 (86%)	98 (72%)	0.06

Continuous data are presented as median (IQR). Categorical data are presented as *n* (%). RBBB, right bundle branch block.

**Table 5:** ECG characteristics in rTOF by type of surgery at different timepoints. Variables with significant differences at more than time point were included in the table.

Variable/timepoint	Surgical strategy	1	2	3	4	5	6	7
QTc (msec)								
	VSR	412 (400, 427)	451 (430, 467)	423 (404, 441)	394 (375, 414)	452 (437, 484)	385 (312, 415)	366 (352, 381)
	TAP	392 (385, 407)	423 (405, 445)	427 (394, 452)	394 (380, 433)	440 (423, 462)	375 (336, 435)	385 (347, 410)
	TAP + M	404 (386, 420)	448 (417, 467)	443 (419, 462)	407 (388, 428)	484 (462, 500)	388 (370, 434)	385 (36, 404)
	P	0.0001	0.0022	0.0011	0.0397	0.0025	0.7262	0.8205
JTc (msec)								
	VSR	342 (329, 361)	355 (341, 369)	325 (311, 342)	290 (271, 306)	318 (304, 349)	253 (178, 290)	263 (240, 286)
	TAP	327 (316, 339)	335 (326, 353)	334 (316, 353)	293 (278, 316)	317 (296, 344)	250 (234, 302)	324 (298, 344)
	TAP + M	337 (323, 352)	351 (331, 366)	338 (329, 351)	294 (281, 318)	341 (327, 363)	259 (237, 291)	327 (307, 351)
	p	0.0001	0.0022	0.0014	0.3079	0.0159	0.8237	0.1243

Continuous data are presented as median (IQR).

VSR, valve sparing repair; TAP, trans-annular patch; TAP + M, trans-annular patch with monocusp reconstruction.

Time points: 1. 1. Prior to primary repair, 2. At discharge from primary repair, 3. 12 ± 6 months after primary repair, 4. 6 years ± 1 year after primary repair, 5. Prior to pulmonary valve replacement, 6. At discharge from pulmonary valve replacement and 7. At 12 months +/ 6 months after pulmonary valve replacement.

For QTc there were significant differences between the three groups prior to primary repair, postop from primary repair, at 1 year follow up, 6 year follow up and prior to PVR (Figure 5A). Overall, the group undergoing TAP had shorter QTc compared to TAP + M whereas the group who underwent valve sparing repair initially had longer QTc, but then decreased to similar duration as the TAP group by 1 and 6 years after primary repair. There were no differences between the groups after PVR and at 1 year after PVR.

For JTc there were significant differences between the three groups from pre-repair until 1 year after primary repair with the shortest duration in the TAP group at the first two time points (*p* = 0.004 and *p* = 0.005 respectively) (Figure 5B). There were no differences at 6 years after primary repair but prior to PVR there was a significant difference with the longest JTc in the TAP + M group and similar JTc in the VSR and TAP groups (*p* = 0.02). At 1 year post PVR the JTc was shortest in the VSR group but this did not achieve significance (*p* = 0.12).

## 4 Discussion

This was a retrospective review of longitudinal ECG changes following repair of Tetralogy of Fallot in a cohort of infant repair showing that different parameters change differently over time and following PVR which indicates that they represent different aspects of remodeling. Demographical data showed that a significant number of patients were born prematurely (20%) but the incidence of prematurity was similarly distributed among the three surgical strategy groups. Valve sparing repair was the most common strategy (41%) followed by TAP + M reconstruction (37%). The group undergoing repair with only TAP was the smallest in number but also the oldest, reflecting a change in surgical strategy over time (follow up 16.4 years vs. 12.1 for VSR and 10.5 for TAP + M respectively; *p* = 0.0001). Our hospital started performing repairs combining TAP with monocusp starting mid 2000s and fewer isolated TAPs.

Other centers have also reported a change in surgical strategy over time with some also performing more valve sparing repairs vs. other centers performing more TAP unlike our data (21, 22). In our cohort, there is less need for PVR in the group with VSR whereas the rate at 10 years is greatest for those undergoing TAP + M (5% vs. 24%, *p* = 0.0006). The rate of reoperation is highest in the group undergoing TAP (38% vs. 16% in the VSR group and 28% in the TAP + M group; *p* = 0.05). The median age of reintervention and PVR is quite low in this cohort. With longer follow-up, more patients are expected to need reoperation and PVR leading to an increased median age of reintervention and PVR.

Burden of arrhythmias was low in this large cohort with adolescent follow up (9 cases) with majority being perioperative. This is consistent with arrhythmias occurring late in rTOF, in particular in the 5th decade of life (23, 24).

### 4.1 PQ interval

The PQ interval represents time for atrial depolarization and delay in the AV node. It showed a modest increase at the time of primary repair from 112 msec (IQR 102, 124) to 120 msec (IQR 103, 134; *p* = 0.004) but was at its highest prior to PVR at 141 msec (IQR 123, 156) and decreased early after PVR to 129 msec (IQR 121, 141; *p* = 0.0005). With sudden increased volume load of the right ventricle, right atrial pressure increases which may explain the increase at primary repair, peaking prior to PVR and the decrease with PVR as this acutely removes the volume load. Massin et al. have also shown an increase in PQ interval over time whereas Kimura et al. not only showed similar pattern with increased PQ interval over time, but also a decrease with PVR, correlation with RV size and function as well as PQ interval >200 as a risk of ventricular arrhythmias (12, 14). Prolonged PR interval also identifies rTOF patients at risk of atrial tachyarrhythmias (13). Even though the PQ interval prolongs in our cohort, most patients don't reach severe levels. PQ interval increases with age, which explain some of the increase. However, the sudden change seen at the time of primary repair and directly after PVR cannot be explained by this. Reported normal values of the mean PQ interval at the corresponding age after primary primary repair and prior to PVR are 98–108 msec and 126–131 msec respectively compared to 120 msec and 141 msec in our cohort (25–27). These are the two timepoints when there either is a sudden increase in volume load or largest volume load of the right ventricle respectively. PQ interval may also be impacted by heart rate, in that it shortens with increased heart rate. However, we have multiple time intervals (between time point 4 and 5, and 5 and 7) where the heart rate is quite similar but there is a change in PQ interval, thereby having another explanation for change (28).

In our study the temporal pattern of PQ interval best resembled the expected volume load of the RV in rTOF.

### 4.2 QRS and QRS dispersion

As expected, QRS duration increased dramatically at the time of primary repair from 66 msec (IQR 61, 73) to 96 msec (IQR 82, 105; *p* < 0.0001), increased consistently thereafter and was highest pre-PVR at 129 msec (IQR 118, 145). There was a significant, albeit small decrease directly following PVR to 120 msec (IQR 109, 132; *p* = 0.0002) but no further decrease 1 year post PVR. QRS duration >170 msec in children is associated with risk of ventricular tachycardias (VT) but as a whole, the QRS duration is significantly shorter in our cohort (10). The relationship between QRS duration and RV size and function has been questioned with varying results and may be less prolonged with the transatrial approach of primary repair (29–31). This is consistent with the findings by Lubocka et al, where the correlation with RV function only is seen with patients with early repair (29).

QRS duration may have multiple causative components, of which RV volume is one (32). The slight early reduction in QRS post PVR may be due to the immediate volume unload from the procedure but there is remaining injury to the conduction system (incl. RBBB) and underlying fibrosis and maladaptation of the ventricle, which is not reversed by this procedure. This is supported by our findings that QRS does not decrease further at one year after PVR. Similar temporary effects on QRS after PVR has been described (33).

In contrast, other markers may be more representative of maladaptation and fibrosis. In our study QRS dispersion shows a small increase over time from 10 msec prior to primary repair to 20 msec pre PVR, but no further significant increase after PVR. This may be due to localized injury and fibrosis, which leads to heterogeneity of depolarization and would therefore not decrease with PVR, especially in short term. QRS dispersion has been found to be associated with increased mortality in patients with congestive heart failure and a marker of impaired function and arrhythmogenic RV cardiomyopathy (34–36). In rTOF, dispersion is greater than in healthy controls but there has been no association found with ventricular size or mass (37). QRS dispersion >35 msec was associated with malignant ventricular arrythmias in rTOF (19). QRS dispersion in our cohort is however lower than reported in older cohorts of rTOF as well as other diagnoses (19, 35). This may represent less damaged myocardium with infant repair as well a younger study population in our study.

### 4.3 QTc, QTc dispersion and JTc

QTc is a composite marker of depolarization and repolarization of the ventricles. In our study QTc increased with primary repair but decreased after 1 year and even further 6 years after primary repair. QTc was the longest prior to PVR being 460 msec (IQR 439, 484), but there was a significant regression after PVR to 384 msec (IQR 342, 434; *p* < 0.0001) and then unchanged. Berul et al. found that a QTc was longer in patients with rTOF who developed VT but inferior as a marker than QRS duration (10). Van den Berg et al. described that QTc was prolonged in patients with rTOF but did not change with exercise (38). Furthermore, it was associated with worsening RVEF and larger RV volume. Our data suggests that QTc does not represent RV volume, as it decreases 1 and 6 years after primary repair, but rather represents an acute adaptation to volume load, since it acutely increases at the time of primary repair and is at its longest at the time of PVR and decreases with relief of the volume load.

QTc dispersion represents inhomogeneity of depolarization and repolarization of the ventricles. QTc dispersion seems to increase the most at the time directly after PVR but there is also an increase at the time of primary repair and prior to PVR. This pattern suggests that surgery causes QTc dispersion, of which some might be temporary and that an already maladapted myocardium is more sensitive as the increase is most marked at the time of PVR. QTc dispersion has only been studied limited in patients with rTOF. QT dispersion >60 msec is associated with increased risk of malignant ventricular arrhythmias (19). Studies have shown that QTc dispersion is prolonged in rTOF but none have studied it longitudinally (10, 39).

The JTc interval represents ventricular repolarization. There is small increase with primary repair, but it decreases over time and is shortest at 6 years after repair and just after PVR. The explanation for this pattern remains unclear. It could be due to prolonged depolarization, leaving shorter time for repolarization.

### 4.4 Right bundle branch block

RBBB is uncommon prior to primary repair in TOF occurring only in 4% of cases but increases significantly following primary repair to 61% (*p *= <0.001) and increases subsequently through the early postoperative period after PVR (89%). Horowitz et al. showed that RBBB is related to infundibular resection in rTOF and more recently Verzaal et al. showed that the main mechanism is block in the Purkinje system (40, 41). Of interest, RBBB increases after PVR, which could be due to the need to excise additional muscle to accommodate the conduit.

RBBB causes RV dyssynchrony by early activation of the septum, thereby impacting the efficiency of RV contraction (7). With increased QRS duration follows potentially more dyssynchrony and harm whereas those without RBBB have better function and lower volumes (11, 42). In our study, there was only a trend at 6 years after primary repair adding to the complexity of understanding the relationship between injury, adaptation etc.

### 4.5 Fragmentation of QRS

QRSf has been shown to be due to regional delays due to scarring in patients with previous myocardial infarction (43). It has been shown to predict arrhythmias and mortality in rTOF (20, 44, 45). Previous studies have shown only low to moderate correlation with right ventricular size and function (46). Presence of fragmentation varies greatly between studies from 35%–81% (20, 44, 46). In our study most patients developed moderate QRSf over time with the highest risk 1 year after PVR (90%; OR 4.2 vs. prior to primary repair; *p* < 0.001). The discrepancy in incidence of QRSf may be due to differences in morphology and thereby classification of QRSf leading to potential interobserver variability (47). More work is needed to identify the morphology of benign vs. concerning types of QRSf in rTOF.

### 4.6 Impact of surgical strategy at primary repair

There is preoperative heterogeneity in anatomy between those who require TAP or TAP + M as they are more likely to have significant outflow tract obstruction and lower saturations as compared to those who could under VSR, who may have more pulmonary over circulation and higher saturations. This is evidenced by the difference in pulmonary valve *Z*-score. In follow up, the difference in size of scar and risk of residual outflow tract obstruction may also impact the load on the right ventricle. We found however, that the type of surgery at the time of primary repair has limited impact on ECG changes, the effect occurs early and disappears after PVR. It seems that surgery in particular impacts repolarization as differences at more than one time point were seen only in QTc and JTc.

Both QTc and JTc are longest in patients with VSR and TAP + M reconstruction early on (Figures 5A,B) and shortest in the TAP group. QTc and JTc are prolonged early in the time course in the VSR group but with recovery duration is similar to monocusp group. This may be due to a longer incision and subsequent scar in the RVOT needed to accommodate the monocusp valve rather that in a TAP alone. A VSR may have more preoperative volume load (due to less RVOT obstruction) explaining why the QTc and JTc are prolonged early in the time course in this group but with recovery duration is similar to the TAP + M group. Other studies have not found an association between TAP and JTc (48). After PVR there is no difference in QTc or JTc between the three groups possibly due to additional incision performed at the time of PVR leveling out the degree of damage and scarring in the three groups.

We were not able to identify clear and consistent markers associated with PVR. Further study of other ECG based biomarkers are needed or correlation to circulating or imaging biomarkers to better understand the meaning of these changes.

### 4.7 Limitations

This was a retrospective study which has the potential for selection bias for those with more severe disease having more ECGs available. The national registry of CHD in Sweden is comprehensive and allows for follow up of outcomes unless a patient leaves the country. As congenital cardiac surgery is centralized to two centers in Sweden, one being Lund, we expected that our material represents a wide variety of backgrounds and referral patterns. Even though the measurements were performed by the same person (MB), there may be intraobserver variability in the study and interobserver which can affect generalizability. Numbers for these have already been published with intra- and interobserver variability between 2%–6% for both QRS dispersion (19). Similarly, interobserver variability is reported to be <5% for PQ, QRS and QTc (12). For fragmentation, there may be difference in interpretation depending on reader. However, since our study looked at longitudinal changes, inter-observer variability is less relevant. Manually measuring and calculating non-standard measures such as JTc and dispersion are time consuming to perform in clinical use which may limit their utility in common clinical practice.

There is also a limitation in duration of follow up in the group that did not undergo PVR and needs to be addressed in a future study. The current clinical data from this should allow for identification of an optimal follow up interval to allow comparison with the PVR group. With longer follow up, we expect that the rate of reintervention and PVR to increase. Thereby, parts of the analysis, including the predictive parameters, could be re-analyzed in a larger cohort with longer follow-up, potentially allowing to identify factors of risk.

Our study also only includes the pediatric and adolescent population. It is unclear how these parameters change in adulthood. The cohort is also young and the incidence of arrhythmias beyond the postoperative period are rare. Since malignant arrythmias occur later in life, we are not able to associate our findings with long term complications.

## 5 Conclusions

Adverse ECG changes are frequent in the pediatric and adolescent population of rTOF. QRS duration and PQ interval both seems to follow the expected pattern of progression and recovery of RV volume but previous studies have been more consistent with PQ interval. Since PQ interval is an easy measurement with low interobserver variability, PQ interval is an underutilized ECG marker in rTOF and needs further study to identify cutoff limits (12).

Surgical strategy at the time of primary repair has only limited impact on ECG findings and that too, only early and in repolarization. After PVR, type of primary repair no longer is associated with any difference.

## Data Availability

The raw data supporting the conclusions of this article will be made available by the authors, without undue reservation.
