# Correction: Longitudinal ECG changes in tetralogy of Fallot and association with surgical repair

**DOI:** 10.3389/fcvm.2026.1860461

**Published:** 2026-05-08

**Authors:** Misha Bhat, Torsten Malm, Gunnar Sjöberg, Felicia Nordenstam, Katarina Hanséus, Carl-Johan Rosenkvist, Petru Liuba

**Affiliations:** 1 Department of Pediatric Cardiology, Pediatric Heart Center, Skane University Hospital, Lund, Sweden; 2 Department of Clinical Sciences, Lund University, Lund, Sweden; 3 Department of Pediatric Cardiac Surgery, Pediatric Heart Center, Skane University Hospital, Lund, Sweden; 4 Department of Pediatric Cardiology, Department of Women’s and Children’s Health Karolinska Institutet, Karolinska University Hospital, Stockholm, Sweden; 5 Department of Pediatrics, Kalmar Länssjukhus, Kalmar, Sweden

**Keywords:** tetralogy of Fallot, trans-annular patch, valve sparing surgery, electrocardiography, QRS duration, QTc, fragmentation, monocusp pulmonary valve

There was a mistake in Figure 5 as published. Some of the *p*-values were not included and the figure did not correspond to the figure caption.

The corrected Figure 5 appears below.

The original version of this article has been updated.

**Figure 5:**
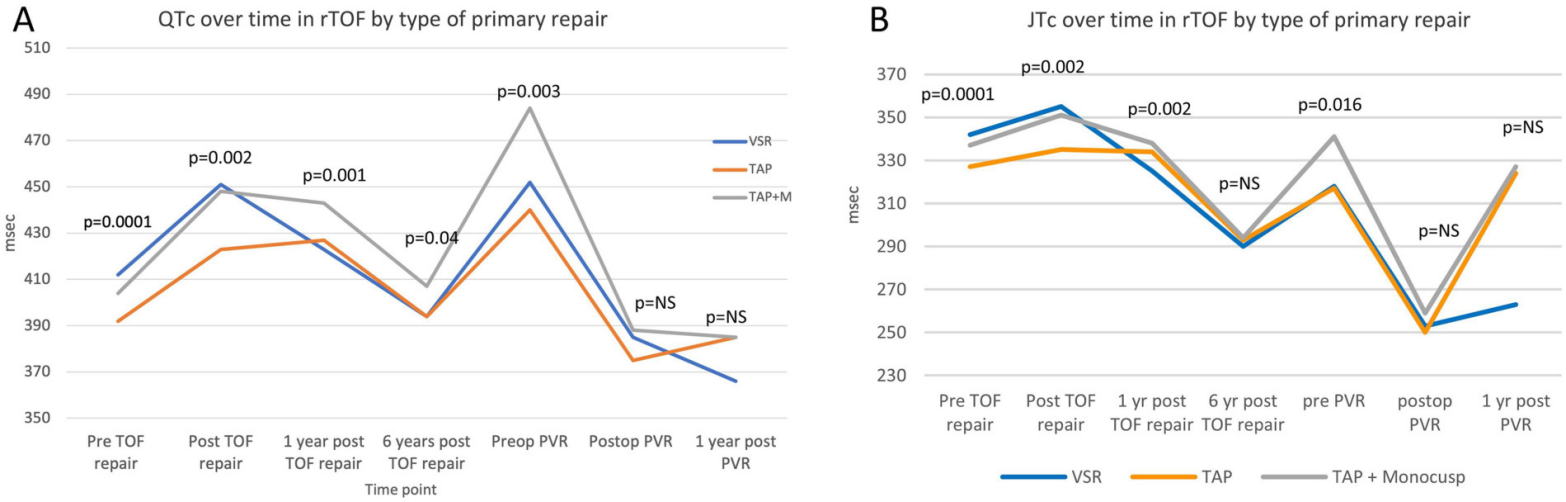
Line diagrams showing comparing QTC **(A)** and JTc **(B)** by type of primary repair. PVR, pulmonary valve replacement; VSR, valve sparing repair; TAP, trans-annular patch; TAP + M, transannular patch with a monocusp reconstruction.

